# Multiresponse Optimization and Environmental Analysis in Direct Recycling Hot Press Forging of Aluminum AA6061

**DOI:** 10.3390/ma12121918

**Published:** 2019-06-14

**Authors:** Nur Kamilah Yusuf, Mohd Amri Lajis, Azlan Ahmad

**Affiliations:** 1 Sustainable Manufacturing and Recycling Technology, Advanced Manufacturing and Materials Center (SMART-AMMC), Universiti Tun Hussein Onn Malaysia (UTHM), 86400 Parit Raja, Batu Pahat, Johor, Malaysia; amri@uthm.edu.my; 2 Department of Mechanical Engineering, Universiti Teknologi PETRONAS, Seri Iskandar 32610, Perak Darul Ridzuan, Malaysia; azlan.ahmad@utp.edu.my

**Keywords:** sustainable manufacturing, direct metal recycling, hot press forging (HPF), aluminum AA6061, life cycle assessment (LCA), response surface methodology (RSM)

## Abstract

Ecological manageability in manufacturing these days is a dire and exceptional issue and the principle concerns are identified with increasingly proficient utilization of energy and materials. Recycling can save a large amount greenhouse gas emissions, particularly in the case of aluminum. The parameter on the innovative technique on the direct recycling was investigated by employing design of experiments, via hot press forging process (DR-HPF). Thus, reutilizing of aluminum chips AA6061 with full factorial 3^2^ design of experiment comprising a variety of working temperature and holding time were employed. Central composite design (CCD) was applied to outline the experiments towards evaluating the influences of the hot press forging parameters to the three responses; ultimate tensile strength (UTS), elongation to failure (ETF), and global warming potential (GWP). In conjunction with this, the environmental impacts associated with DR-HPF process are evaluated alongside the resultant conventional recycling (CR) by using re-melting route as indication. Experimental measurements, literature analysis and industrial data were merged to acquire the analysis of aluminum recycling life cycle. Clear conclusions were successfully drawn through the attained results on the outlook proposed by solid state direct recycling for the purpose of reducing the environmental effects by taking material and energy conservation as one of the most essential impacting factor. The global warming potential of a DR-HPF route gives a significant environmental impact where it is reduced up to 69.2% in comparison to the conventional (melting) routes.

## 1. Introduction

Climate change is regarded as one of the most prominent and challenging environmental problem and its mitigation is a major objective in environmental management. Researchers have affirmed that the Earth’s environment and seas are warming steadily because of human action [1]. There is a developing global logical accord that this expansion has been brought about by human action, principally the consuming of petroleum products for such exercises as creating power. Since the pre-modern period, climatic convergences of CO_2_ have expanded by almost 30% and methane (CH_4_) fixations have dramatically increased [2]. Different modern procedures represented roughly 14% of the complete CO_2_ discharges and 20% of the highest ozone harming substance emanations in 2010 [3]. The aluminum industry alone is accountable for approximately 1% of global greenhouse gas emissions [4]. Conferring to [5], the assembling part, which convenes at the center of the innovative cornucopia, must be made practical to save the restricted requirement of living achieved by developed social orders and to encourage creation of social orders to accomplish and keep up a similar way of life. Therefore, the need of decrement of energy consumption in industrial processes, as well as transportation and production engineering, has become a major factor in the modern industrial world.

Aluminum is one of the most important commodities of our modern society as it is a critical component in a wide range of primary industries that include construction, transportation (aerospace and automotive), healthcare, and food packaging. Moreover, with the shift to a low carbon economy, the structural strength and lightweight features of aluminum have a major impact on energy savings and reduction in greenhouse gasses (GHG) emissions through their role as structural composites in the automotive and aerospace industries. Secondary aluminum is aluminum that is derived from scrap aluminum by recycling processes. Scrap metal can be classified as new scrap (aluminum left over from manufacturing processes) or old scrap (aluminum left over following consumer use) [6]. Reuse is, as yet, a standout among the most encouraging approaches to add to the constraints of the ecological effect of aluminum creation. Undoubtedly, auxiliary aluminum creation from scrap by conventional re-dissolving requires substantially less energy than essential generation, by and large it is conceivable to express that optional aluminum generation requires just about 10% of the energy required by essential creation (the entire creation course). Current practices of recycling aluminum in most industries are using a melting technique to produce a secondary ingot by controlling the composition of alloys to match the standardized grades. A recent study indicates that by 2030, excessive amounts of aluminum alloying elements will lead to non-recyclable scrap amounting to 6.1 megatons [7]. Since the elimination of alloying component amid re-dissolving is hazardous for the majority of the components and in this way, unique methodology, concentrating on meltless reusing of aluminum scrap can give noteworthy ecological advantages by maintaining a strategic distance from metal failures amid re-softening. In spite of that, the aluminum reusing process is as yet an energy escalated one, and the general energy productivity is low [8]. Substantial quantities of both material and energy can be expend through avoiding re-melting which subsequently reducing the overall effects on the ecological impact by decreasing the utilization of aluminum. Innovative approaches have been applied to the recycling of aluminum chips by employing solid-state recycling techniques. Initially, the solid-state recycling techniques utilized the powder metallurgy processes [9] followed by [10]. However, the solid-state technique has recently enhanced by utilizing direct recycling (DR) technique, eliminating the pre-process, such as ball-milling, to produce fine granulated particles. As the preliminary step is eliminated, the process is called direct recycling. This chain of procedures requires just 5–10 % energy of that required for the regular procedure chain that incorporates a re-softening advance of the piece to deliver new extrusion billets [11]. Haase et al. [12] utilizes the ECAP (equal channel angular pressing) incorporated expulsion master cesses to combine chips into billet. An immediate screw expulsion strategy was displayed by Widerøe et al. [13] where this procedure acquaints rotational development with the piece compacting and expels profiles in a single step. Paraskevas et al. [8] proposed the use of spark plasma sintering (SPS) as an innovative solid state recycling technique. The study that utilized DR using hot forging process shows that recycled aluminum exhibits good strength and plasticity potential, proving that the solid-state technique with hot forging is an acceptable alternative for recycling aluminum alloy chips. Additional research in this particular study area is highly recommended for further environmental conservation [14,15]. Hot press forging (HPF), in any case, has shown itself to be a promising elective technique for reusing aluminum as the loss from the machining procedure shows magnificent potential as far as quality and pliancy [16,17,18,19,20,21].

The increasing discernment on the significance of ecological security and the conceivable effects related with items produced has expanded the enthusiasm for the advancement of techniques to all the more likely understand and decrease these effects. Beyond conservational issues, monetary concerns likewise add to manageability and sustainability of the methods. Life cycle assessment (LCA) is one of the method that is currently explored [22]. LCA is one of the extensively used and internationally accepted methods for evaluation of the environmental impacts of products and system which able to efficiently assess the ecological results and to break down the trades that occur in nature and are identified with the analysed item or procedure. Indeed, the method offers a broad potential for industrial development of new techniques, especially in the recycling of aluminum (solid-state) as environmentally benevolent options from the existing practices [23]. Also, the classification of feasible procedures for metal molding technologies, which advances energy and resource-efficient methodologies for aluminum-based part assembling is an examination subject that ought to be investigated with some earnestness [24]. Duflou et al. [23] analyze the natural execution of three imaginative strong state reusing procedures with the ecological effect of the traditional re-dissolving procedure. The paper validates the noteworthy ecological standpoint reachable through utilizing the inventive reusing systems. LCA studies on metal waste recycling are very scarce and almost no database recordings exist so far for the recycling of aluminum by using a hot press forging process. This paper revealed the environmental study of solid-state recycling (hot press forging technique) incorporated with LCA by using Design Expert software.

On top of that, many research works have been accounted for the utilization of response surface methodology (RSM) on improving of process parameters by attractive quality approach on metal forming and machining process [25,26,27]. Instead of one factor at a time (OFAT), RSM has been widely employed as it is time saving and more cost effective [28]. For that reason, RSM was employed in this study to optimize the parameter effects on direct recycling aluminum chip AA6061 with considered the functional performance and environmental benefit by setting the desirability to maximize the UTS and ETF, whereas minimize the GWP. With the optimum parameter of direct recycling hot press forging (DR-HPF) that suggested from RSM analysis, the present paper continued to estimate and compare the environmental impacts of this technique with conventional recycling (CR) route through LCA. The paper focused on identifying the available opportunities in alternative recycling routes for production waste with higher recycling values. Hence, this approach seeks to minimize the need for primary material flows and in turn reduce the adverse environmental impact. The concerns for investigation include the energy and CO_2_ outflows life cycle investigation of a genuine contextual analysis. Specifically, the end-of-life recycling approach was reviewed with special reference to aluminum.

## 2. Materials and Methods

### 2.1. Hot Press Forging

**Figure 1:**
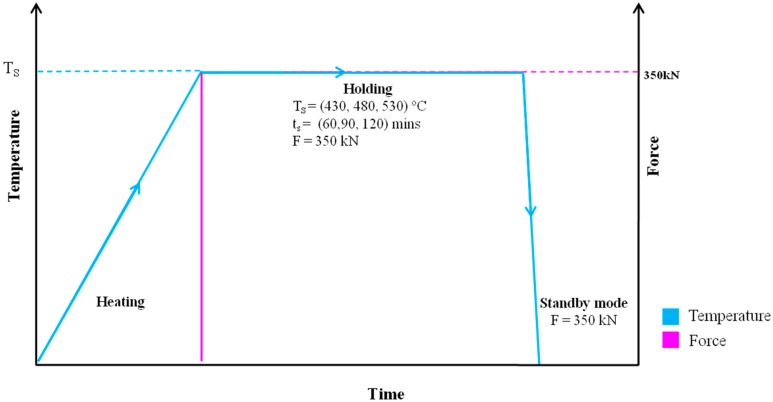
Hot press forging diagram.

Aluminum billet with series AA6061-T6 (Sigma-Aldrich, Saint Louis, MI, USA) was processed to deliver medium size aluminum chips. The processing occurred in the Sodick-MC430L (Baginton, Coventry, UK) fast machining, with the cutting rate, v of 110 m/min, feed rate, f of 0.05 mm/tooth and the profundity of cut of 1.0 mm. A cleaning procedure in acetone (C_3_H_6_O) solution starts as soon the chip leaves the processing machine. The drying procedure at that point experiences for 30 min in warm drying broiler at 60 °C. The cleaned aluminum chip was filled the form and the dive is fixed appropriately. Hot press manufacturing process executed for 60, 90, and 120 min at 430, 480, and 530 °C with four pre-compacting cycles. The force was set to steady at 350 kN (around 35 tons). The general chart of the hot press operating procedure is shown in Figure 1. The forming procedure beforehand brought about arrangement of a standard example for ductile testing (ASTM E8-M). A tractable test at room temperature were led on a universal testing machine (UN-7001-LS, GOTECH Testing Machines Inc., Taichung, Taiwan) at the test speed of 0.50 mm/min.

### 2.2. Experimental Design

Response surface methodology (RSM) utilizing a succession of planning tests was applied in this study to acquire ideal reactions. RSM is a strategy for improvement in the utilization of measurable and numerical systems helpful for creation, improvement, and enhancement processes. Focal composite-plan (CCD) was picked as the RSM structure that is helpful for exploring the quadratic impacts. The Design Expert 8.0 program was utilized to build up the exploratory arrangement for RSM. A numerical model as different relapse conditions of the second request reaction surface with the best fittings was created. In this paper, RSM was utilized to explore the connection between three reactions (UTS, ETF, and GWP) and the two most vital parameters are temperature and holding time, just as to improve the applicable states of the parameter to anticipate the best reactions. Table 1 exhibits the provisional model of parameters.

The experiments were improved with replications at the outline focus to survey the immaculate mistake and were conveyed in randomized request, as required in many plan systems. A coded plan variable was utilized because it quantifies the impact of changing on every outline calculate over a one-unit interim and it is additionally dimensionless. Coded designs can be directly comparable to the model coefficient magnitudes and the relationship was bound as in Equation (1) [29].

(1) $$C=\frac{X-\frac{\left(A_{l}+A_{h}\right)}{2}}{\frac{\left(A_{h}-A_{l}\right)}{2}}$$

where *C* is the coded design variable; *X* is the actual intended magnitude; *A_l_* and *A_h_* are the actual low and high magnitude respectively. Since there are just three dimensions for each factor, the proper model for foreseeing the ideal conditions is the accompanying quadratic model structure as shown in Equation (2) [30].

(2) $$y=\beta_{0}+\overset{k}{\underset{j=1}{\sum }}\beta_{j}x_{j}+\overset{k}{\underset{j=1}{\sum }}\beta_{jj}{x^{2}}_{j}+\underset{i}{\sum }\overset{k}{\underset{<j=1}{\sum }}\beta_{ij}x_{i}x_{j}+\epsilon$$

where *y* is the response; *x_i_* and *x_j_* are the factors; *β*_0_ is a constant coefficient; *β_j_*, *β_jj_*, and *β_ij_* are the interaction coefficients of the linear, quadratic, and second-order terms, respectively; *k* is the number of studied factors and *ϵ* is the error. The fitted polynomial condition is communicated as surface and form plots so as to picture the connection between the reaction and exploratory dimensions of each factor and to find the ideal conditions [31,32]. Subsequent to optimization, model equation adequacy for predicting the optimum response values was substantiated with experimental results.

The conditions were assessed for every reaction by methods for various straight degeneration study. The noteworthy terms in the model were found by analysis of variance (ANOVA) for every reaction. The probability level is used to measure the significant at which it is calculated from the data and the result is less than 5%. The determination coefficient R^2^ is then used to resolve the model adequacies, adjusted determination coefficient (adjusted R^2^), anticipated R^2^ and expectation mistake aggregate of squares (PRESS). In the wake of playing out the model fitting, residual investigation was led to approve the suspicions utilized in ANOVA [25]. The subsequently fitted polynomials amplification and minimization was typically achieved by allure quality capacity method. The allure quality capacity approach is a standout among the most often utilized multi reaction enhancement procedures immediately. Allure capacity is a target that reaches from zero outside of the points of confinement to one at the objective. The attributes of an objective might be changed by conforming the weight or significance. For a few reactions and variables, all objectives must be improved all the while into one attractive quality capacity. Equation (3) depicted the coinciding objective function, which is a geometric aptitude of all altered responses.

(3) $$D={\left(d_{1}\times d_{2}\times \ldots \times d_{n}\right)}^{\frac{1}{n}}={\left[\overset{n}{\underset{i=1}{\prod }}d_{i}\right]}^{\frac{1}{n}}$$

where *D* is overall desirability and *n* is the responses number in the measurements. In the event that any of the reactions or elements falls outside their attractive quality range, the general capacity ends up zero. For concurrent streamlining, every reaction must have a low and high value allotted to every objective.

### 2.3. Life Cycle Assessment Methodology and Scope Definition

Life cycle assessment (LCA) is one of the generally utilized and globally acknowledged strategies for the assessment of the ecological effects of items and a framework which is designed to deliberately gauge the ecological results and to dissect the trades that happen with the earth and are identified with the inspected item or process. This section discussed in the evaluation of carbon footprint by means of the global warming potential value for each parameter setup consists of different operating temperatures and holding times for the hot press forging process when recycling AA6061 chips. The method and steps in this analysis complied with the ISO14040 and ISO 14044 standards.

System boundaries of the proposed approach are the production waste and the useful output of the processes, compressing the waste, and by-product streams arising from production. The LCA model was made by utilizing the SimaPro 8.0.5 programming (PRé Consultants B.V., Amersfoort, Netherlands) life cycle appraisal. The databases enclosed in the SimaPro programming provide the Life Cycle Inventory (LCI) information of the crude and procedure materials utilizing out of sight framework. The information for customary method is gathered by the Ecoinvent database joined with distributed information from writing.

#### 2.3.1. Input Data Inventories

The details of each procedure in collective with their key inventories sources and data are as shown in Table 2. Required settings and measurement for hot press forging process are included in Table 3. The mean value for energy consumption per unit mass of the hot-pressed profile is between 25.10–53.92 kWh/kg. The ReCiPe method [33] was chosen for the life cycle impact assessment (LCIA). The measurement of the midpoint indicator for global warming potential (GWP) is an extensively acknowledged [1] technique employed to calculate lengthy period CO_2_ corresponding emissions. This method changes all emissions to 100-year CO_2_ equivalents. The results for related gasses to aluminum process (European Aluminum Association, 2008) are accumulated into a single unit in terms of their influence as carbon dioxide equivalents from [1]. Five related gasses are carbon dioxide (CO_2_), methane (CH_4_), sulphur dioxide (SO_2_), nitrous oxide (N_2_O), and perflurocarbon (PFC-14). CO_2_ gasses were mostly released in both routes whereas, N_2_O and PFC-14 not applicable from this process. This GWP values were carried forward to develop a model and optimize the effect of hot press forging parameters over the mechanical properties responses and GWP by employing response surface methodology (RSM).

#### 2.3.2. Life Cycle Assessment (LCA) Methodology

**Figure 2:**
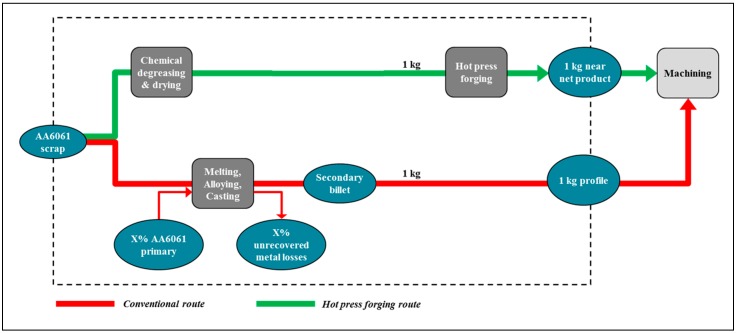
System boundary of the LCA comparison for the different recycling routes.

Since a similar LCA analysis was aimed, 1 kg of crude material in prismatic close net shape type of DR-HPF process was contrasted with a billet of a similar weight acquired after re-liquefying, 1 kg of crude material in a kaleidoscopic close net shape type of DR-HPF process was contrasted with a billet of a similar weight acquired after re-liquefying. System boundaries of the proposed approach is the production waste and the useful output of the processes, compress the waste and by-product streams arising from production. In order to validate this concept, this research contains a relative examination of an elective material reusing course, beginning from indistinguishable waste materials from the CR route, analyzing the technical feasibility as well as the possible material and energy savings. The LCA show was made utilizing the SimaPro 8.0.5 programming forever cycle appraisal. The databases contained in the SimaPro programming give the LCI information of the crude and procedure materials utilized out of sight framework. The information for ordinary strategy is gathered by the Ecoinvent database joined with distributed information from writing. The secured procedure ventures for the distinctive reusing courses appear in Figure 2, with a sign of the mass transitions relating to the picked correlation bases. Compound degreasing and drying just as scrap cold compaction were likewise considered. The substitution methodology, recommended by the European Aluminum Association [6], is ensued in this LCA investigation to adjust the un-recouped metal misfortunes. Thus, metal failures should be offset with essential Al. This methodology is legitimate since the accessibility of aluminum scrap is constrained and the interest surpasses the advertising [34].

The details for each process with their key LCI data and findings were as shown in Table 4. Where accessible effects related with properly recorded procedures, similar to essential aluminum creation, were acquired from the Ecoinvent 3.1 database PRé Consultants B.V.Company, Amersfoort, Netherlands). The medium voltage power mixture (worldwide normal) for the aluminum processes was utilized. A closed alloy AA6061 recycling loop was considered for all the routes, avoiding down-cycling or compositional corrections during melting. Measurement data for hot press forging route is collected by in-house analysis whereas, the data for long-established method is gathered through the Ecoinvent database merged with available reports from the literature. The mean value for consumption of energy for every unit mass of the hot-pressed profile is between 45.01 kWh/kg.

The life cycle impact assessment stage is conducted by using the ReCiPe characterization model. This model takes the given life cycle stock and changes this information into marker scores for the distinctive effect classes. The ReCiPe technique offers the likelihood of deciding the ecological effect inside three endpoint classes. The midpoint classes speak to an issue situated methodology where the effect classifications speak to various sorts of natural pressure. The endpoint classifications result in a concern for ecological outcomes, by gathering the midpoint classes into three endpoint classes addressing natural harms. This strategy is consequently called a harm arranged methodology. In this paper, the endpoint classifications were utilized, since they are easy to interpret, but each impact category was also discussed for robustness. The results of the LCIA will be exhibited and clarified. The outcomes are given per utilitarian unit, being a measure of natural effect per kg of aluminum compound. Additionally, the generally-acknowledged midpoint marker of global warming potential (GWP) determined dependent on the [1] technique was utilized to register long haul CO_2_ comparable outflows. This technique changes over all outflows to 100 years CO_2_ reciprocals.

## 3. Results and Discussions

### 3.1. Response Surface Methodology

In this paper, the accompanying two freedom factor parameters were picked for the investigation: operating temperature, T_s_ as A and holding time, t_s_ as B. The scopes of these parameters were chosen dependent on starter tests directed by utilizing two factors at any given moment approach. Eleven tests were directed as appeared Table 5 which gives the dimension of different parameters and their assignment.

The analyses were upgraded with repetitions at the design center to measure the immaculate error and were conveyed in an arbitrary order, as needed in most of the design procedures. A coded design variable was used because it measures the effect of changing on each design factor over a one-unit interval and it is also dimensionless. Coded designs can be directly comparable to the model coefficient magnitudes and the relationship was bound in an equation. Due to the fact that for each factor, there are solely three levels, the fitting model to forecast the ideal stipulations is the following quadratic model form as shown in Equation (2). The response parameters in the present paper were ultimate tensile test, UTS (MPa); elongation to failure, ETF (%); and global warming potential, GWP (kg CO_2_-eq/kg) to optimise the pertinent stipulations of the parameter to predict the best responses as shown in Table 6. Analysis of variance (ANOVA) was executed for this purpose.

#### 3.1.1. Analysis of Ultimate Tensile Strength

So as to guarantee that it is an appropriate model, test for the criticalness of the relapse display, test for centrality of individual model coefficients, and test for absence of-fit ought to be performed. An ANOVA in Table 7 is generally used to condense the tests performed.The model F-estimation of 172.65 infers the model is noteworthy. There is just a 0.01% shot that a model F-value this substantial could happen because of commotion. Estimations of Prob > F under 0.0500 demonstrates show terms are noteworthy. For this situation A, B, A^2^ is noteworthy model terms. Qualities more significant than 0.1000 demonstrate that the model terms are not huge. The lack of fit F-estimation of 12.13 infers that there is a 7.71% shot that a lack of fit F-value this expansive could occur due to distrubance. These significant effects, in descending order, are factor A (operating temperature), the quadratic effect of factor A (operating temperature).The other model terms are said to be not critical. The fit synopsis prescribed that the quadratic model is factually huge for investigation of UTS. The consequences of the quadratic model for UTS as ANOVA are given in Table 7.

To make the quadratic model UTS appropriate, the non-significant terms are eliminated from pooling process. By selecting the backward elimination procedure to automatically reduce the terms that are not significant, the resulting ANOVA table for the reduced quadratic model for UTS is shown in Table 8. The model F-estimation of 284.51 suggests the model is critical. The lack of fit F-estimation of 10.44 infers there is a 8.97% possibility that a lack of fit F-value this vast could happen because of clamor. Not huge absence of fit is great where it makes the model to fit. The value R^2^ and balanced R^2^ are over 95%. This implies relapse show gives a great clarification on the connection between the autonomous (factors) and the reaction (UTS). The related *p*-value for the model is lower than 0.05 (95% certainty) demonstrates that the model is viewed as measurably noteworthy [27]. The absence of-fit term is not noteworthy as it is wanted. Furthermore, factor A (working temperature) and factor B (holding time), and the second request term of factor A (working temperature) has a noteworthy impact. The reduced model results implied that the model is significant where *R*^2^ and adjusted *R*^2^ are 99.19% and 98.84%, respectively, lack of fit is no significant (*p*-value is less than 0.05). The value of predicted *R*^2^ is 0.9749.

Note that the residuals are falling in a straight line, which implies that the errors are normally scattered. Besides, each observed regard is differentiated and the foreseen values are recorded. It tends to be observed that the relapse is fitted with the observed values.After pooling the non-noteworthy terms, the last reaction condition for UTS is given as follows:

(In coded factors)

UTS = +76.67 + 112.88 A + 18.70 B + 56.31A^2^ (4)

**Figure 3:**
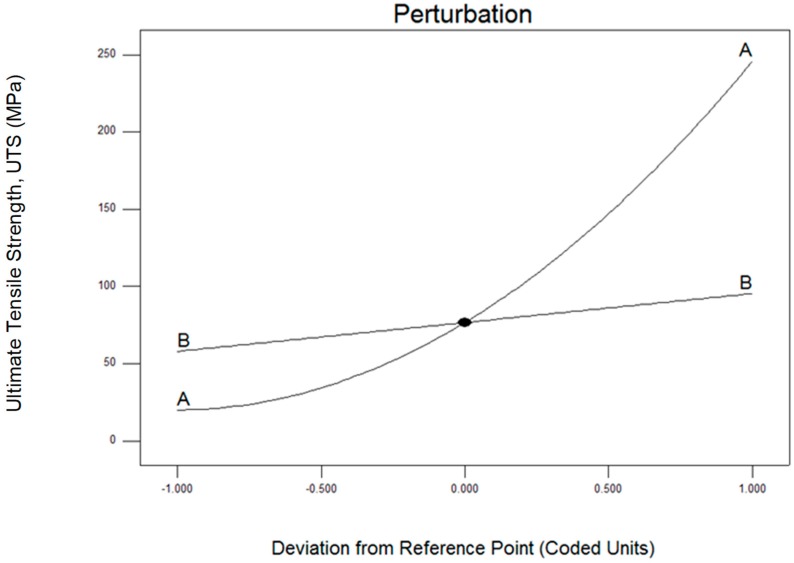
Perturbation plotted graph as a correlation of (**A**) operating temperature and (**B**) holding time for ultimate tensile strength (UTS).

**Figure 4:**
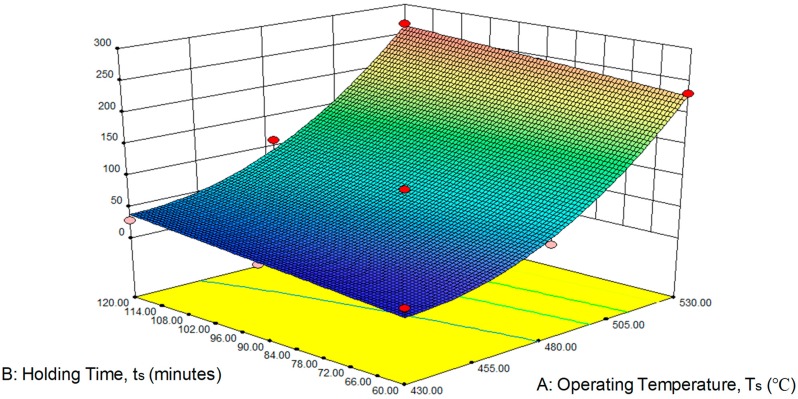
Effect of the (**A**) operating temperature and (**B**) holding time on ultimate tensile strength (UTS).

Figure 3 shows the perturbation plot for UTS. Factor A (operating temperature) gives more influence to factor B (holding time) make a better result on UTS. Figure 4 shows the effect of temperature and preheat time on UTS on 3D graphic. It is clear that the UTS intensifies with the rising of temperature and the increasing holding time. Enhanced UTS can be obtained for a higher operating temperature and followed by increasing holding time rate.

#### 3.1.2. Analysis of Elongation to Failure

A statistical technique was employed to assess the discrepancies concerning two or more means in which the variance analysis were done to implicate the means. Table 9 exhibits the ANOVA table for a quadratic model for elongation to failure, ETF (%) where the value of *R*^2^ is 97.99% and adjusted *R*^2^ is over 95.98%%. This implies that the regression model gives the good relation amid the independent variables (factors) and the elongation (%). The related *p*-value for the model is lower than 0.05 (95% confidence) indicates that the model is statistically substantial. The lack-of-fit term is not significant as it is desired. Furthermore, factor *A* (operating temperature) and factor *B* (holding time), and second order term of factor *A* (operating temperature) have a noteworthy effect. These significant effects, in descending order, are factor *A* (operating temperature), the quadratic effect of factor *A* (operating temperature). The other model terms are deemed to be not substantial.

To correspond the quadratic model to ETF fittingly, the non-noteworthy terms are dispensed with by selecting the regressive end method to naturally lessen the terms that are not critical, the subsequent ANOVA table for the decreased quadratic model for an ETF is shown in Table 10. The model F-estimation of 27.79 infers the model is critical. There is just a 0.03% possibility that a model F-value this extensive could happen because of commotion. Estimations of Prob > F under 0.0500 demonstrates show terms are noteworthy. For this situation A, B, and A^2^ are huge model terms. Values that are more prominent than 0.1000 demonstrate the non-critical model. For elongation, the fit summary recommended that the quadratic model is statistically substantial for analysis. The lack of fit F-value of 9.25 implies the lack of fit is not significant relative to the pure error. There is a 10.04% chance that a lack of fit F-value this large could ensue attributable to the noise. The results of the pooling model imply that the model issubstantial. The prediction R^2^ is 0.7192 is in equitable accord with the altered R^2^ is 0.8893 and lack of fit is inconsequential (*p*-value is less than 0.05).

Notice that the residuals are falling in a straight line, which implies that the blunders are ordinarily circulated. Moreover, each observed value is contrasted and the anticipated value determined from the model. It very well may be seen that the relapse presented genuinely fits well with the observed qualities. In the wake of pooling the non-huge terms, the last reaction condition for lengthening is as follows

(In coded factors)

ETF = +1.73 +4.89 A +2.38 B +1.83 AB +4.21A^2^ (5)

**Figure 5:**
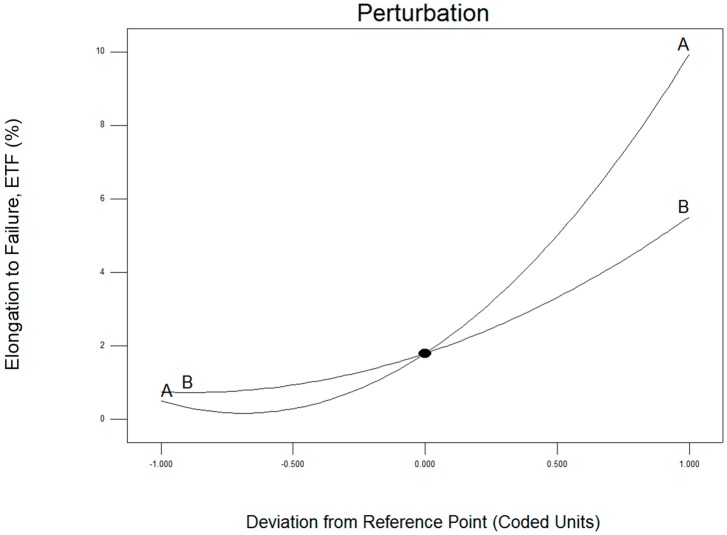
Perturbation plotted graph as a correlation of (**A**) operating temperature and (**B**) holding time for elongation to failure (ETF).

**Figure 6:**
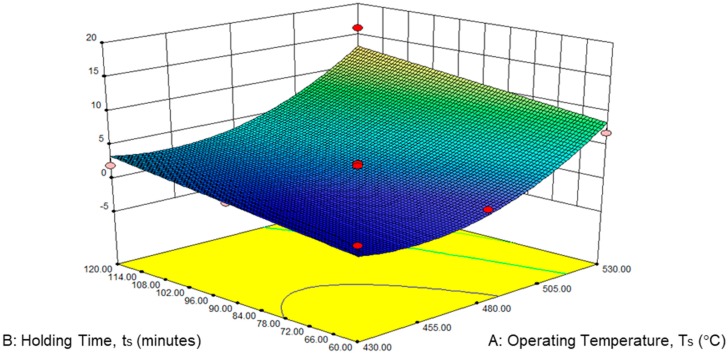
Effect of the (**A**) operating temperature and (**B**) holding time on elongation to failure, ETF.

The perturbation plot for ETF was presented in Figure 5 above. Factor A (operating temperature) shown the dominant on hot press forging process rather than factor B (holding time). The 3D surface graphs for ETF are shown in Figure 6 below. Similarly, the ETF a the tendency to rise, considerably with increases in temperature to 430 °C and 530 °C. In the same way, the rising holding time from 60 to 120 min increased the variation of the ETF. Hence, maximum ETF is obtained at the maximum holding time (120 min) and maximum operating time (530 °C).

#### 3.1.3. Analysis of Global Warming Potential

It was aforementioned that assessment for the regression model significance of the test for noteworthiness on the particularized coefficients model and test for lack-of-fit should be executed. The executed tests were usually summarized using an ANOVA table. Table 11 exhibits the ANOVA table intended for a response surface quadratic model for global warming potential, GWP. The model F-value of 1474.85 implies that the model is substantial. The first order model proposing the linearity is adequate for GWP and further second order model is not necessary. There is just a 0.01% possibility that a model F-value this vast could happen because of noise. Estimations of Prob > F under 0.0500 demonstrate that the model terms are noteworthy. In this case A and B are significant model terms. The lack of fit F-value of 1.11 implies the lack of fit is insignificant corresponding to the pure error. There is a 19.55% chance that a lack of fit F-value this large could occur due to noise. The predicted R^2^ of 0.9958 is in reasonable agreement with the adjusted R^2^ of 0.9966. 

Another essential point, the residuals are falling in a straight line, which implies that the blunders are typically disseminated. Furthermore, each observed value is contrasted and the anticipated value determined. The relapse shows well fits with the observed qualities. The last reaction condition for GWP is given as follows

(In coded factors)

GWP = +30.78 +5.82 A +5.33B
(6)

**Figure 7:**
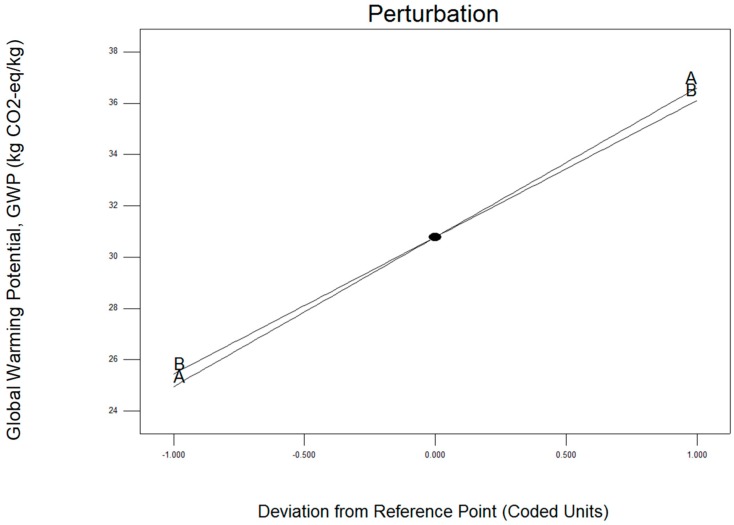
Perturbation plotted graph as a correlation of (**A**) operating temperature and (**B**) holding time for global warming potential, GWP.

**Figure 8:**
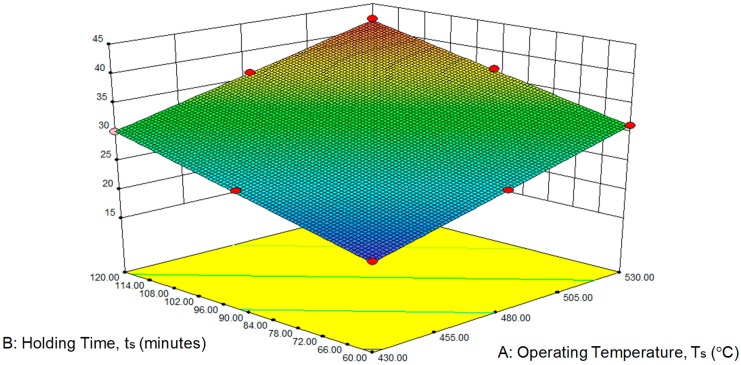
Effect of the (**A**) operating temperature and (**B**) holding time on global warming potential, GWP.

The perturbation plot for GWP was present in Figure 7 above. Factor A (operating temperature) shown the dominance on hot press forging process rather than factor B (holding time). The 3D surface graphs for hardness are shown in Figure 8 below. Similarly, the GWP tends to increase considerably with increases in temperature to 430 °C and 530 °C. In addition, the rising holding time from 60 to 120 min increased the variation of the GWP. Hence, maximum GWP is obtained at the maximum holding time (120 min) and maximum operating time (530 °C).

#### 3.1.4. Optimization Desirability

The aim of this paper was to maximize the mechanical properties UTS (sufficient to reach 241 MPa–ASM theoretical value) and maximize the ETF. It is found that minimizing the GWP is considered as beneficial to the environment. The suggested optimum parameter will be shown in this subsection. Table 12 presents the insinuated solution for desirability to get the desired UTS and maximum ETF while minimize the GWP. With a desirability of 64.2%, the suggested operating temperature is 530 °C for 82.19 min. The predicted UTS is 241 MPa, ETF (10.2093%) and GWP is 35.2106 (kg CO_2_-eq/kg).

**Figure 9:**
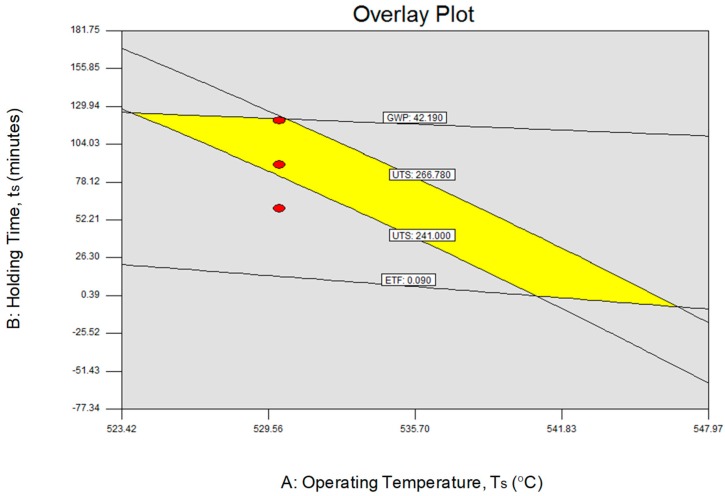
Overlay plot for optimization.

Previously, the analyses discussed were based on the individual responses of UTS, ETF, and GWP. The objective was to obtain the most feasible region of optimization for all responses and factors shall be achieved by overlaid contour plots. This method involves of overlaying individually all responses contour plot and finding the area which makes the best possible value for each of the responses. The overlay plot for restructuring by using RSM are showed up presented by Figure 9. The shaded ranges on the graphical enhancement plot do not meet the assurance criteria. The lines marked the high or low points of confinement of the responses. The yellow colored area marks the feasible range to set the segments to satisfy the necessities on all responses. From this figure, it very well may be assumed that the appealing quality would be met when the UTS, ETF, and GWP mix are inside the yellow territory of the overlay plot.

Since the reaction surface conditions were obtained from a quadratic relapse fit, corroborative tests must be performed to check their legitimacy. The autonomous variable qualities chose for the affirmation test must exist in the extents for which the equations were inferred. To verify the adequacy of the model developed using RSM, two confirmation run experiments are performed and the results achieved are tabulated. Table 13 shows the two confirmation experiments were performed for UTS, ETF, and GWP. Using the point forecast ability of the product empowers the expectation of UTS, ETF, and GWP of the chose investigations complete with the 95% expectation interim. The anticipated qualities and the related expectation interim depend on the models recently created. The anticipated qualities and the real exploratory qualities were analysed and the leftover and the rate mistake determined. Every one of these qualities are recorded in Table 13. The error between predicted and experimental values for UTS and ETF are lying within 2.28% to 4.81% and 13.02% to 19.98%. Respectively, the GWP ranges between 3.18% to 2.84%. Although the percentage of error for an ETF is quick high, but is still within the range of 95% of confidence limit for low and high predicted value. This corroborates the outstanding replicability and recyclability of the experimental conclusions and thus, conclusively, the empirical models developed were relatively correct.

### 3.2. Life Cycle Assessment (LCA)

**Figure 10:**
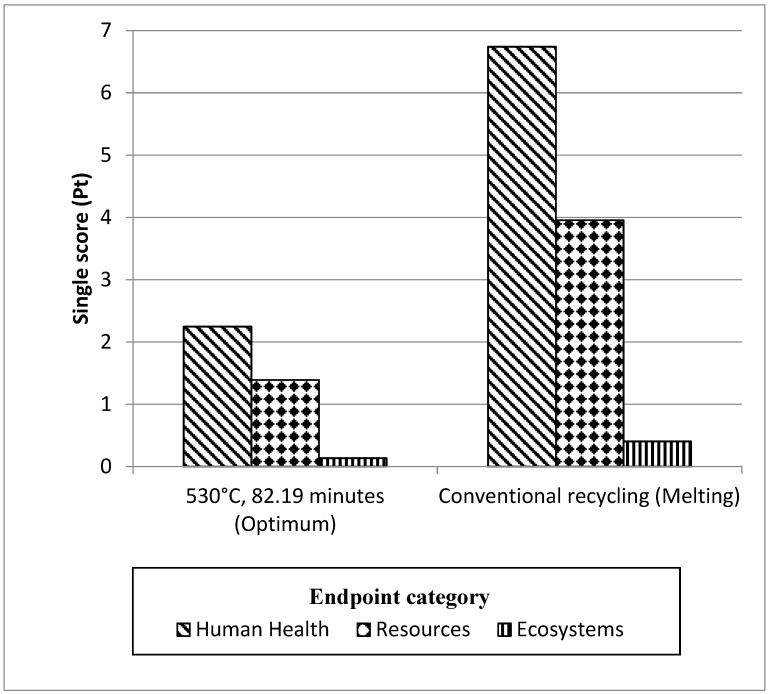
Relative performance endpoints of DR-HPF process and CR of aluminum.

**Figure 11:**
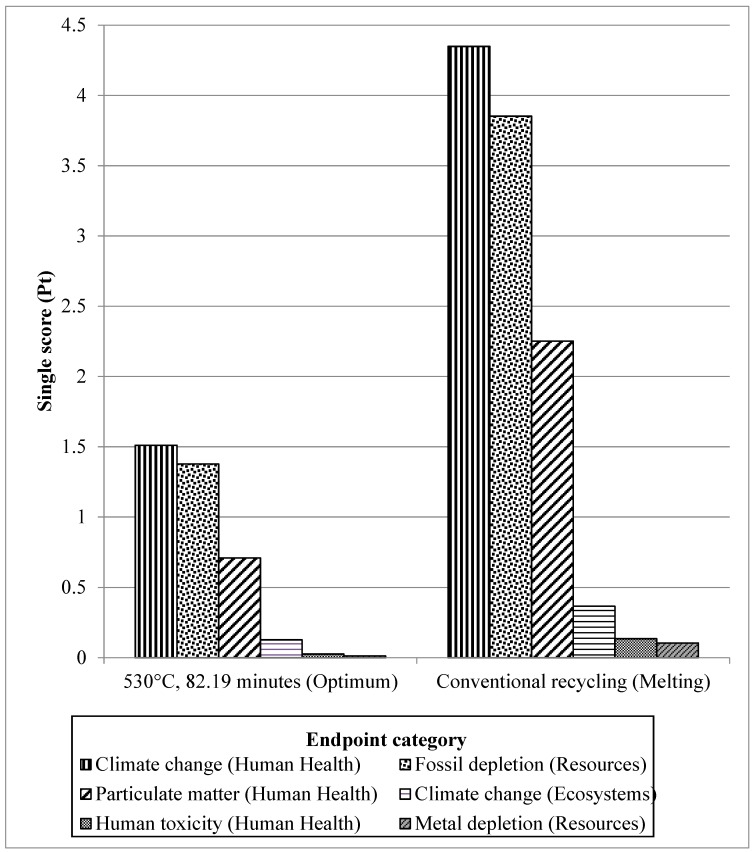
Relative performance (per impact categories) of DR-HPF process and CR of aluminum.

The LCA was performed by employing the ReCiPe Europe H/A procedure [22] and SimaPro 8.0.5. The selected comparison basis was the endpoint level as the influence of the entire endpoint per impact assemblies is accumulated into a single score (Pt). Single score values are the characterization results after normalization and weighting under the conditions. These estimates were revealed in a facile approach from the source of impacts. The results of the LCA for DR-HPF process (530 °C, 82.19 min suggested as optimum from RSM analysis) with CR (melting) were briefly compared aggregated into a single score (Pt) within each impact category as shown in Figure 10. This is an easy way to get a quick overview on how different routes of recycling compare to each other. Figure 10 shows the endpoint categories and to translate these three endpoint categories, Figure 11 shows endpoint per impact categories perform compared to each other, the impact results have are the single score values for the ReCiPe endpoint approach. At the endpoint categories, as in Figure 10, recycling of aluminum has the greatest impact on human health, followed by resources and ecosystem. Figure 11, shows 6 endpoints out of 17 impact categories among the most significant impact values are climate change (human health) and fossil fuel depletion, followed by particulate matter, climate change (ecosystem), human toxicity, and metal depletion.

The best share of effects to the human health endpoint category originate from the after effects of climate change (per impact category). The environmental change class, portrayed by equal ozone harming substances discharge, is currently perceived as being among the most urgent natural issues, shown by the expansive consideration of this issue in global conferences [35]. The release of carbon dioxide from burning of fossil fuels was one of the most significant procedures in aluminum extraction and recycling [36]. The most significant emissions resulting from the aluminum CR process are emissions released into the air. These include dust and smoke, metal compounds, organic materials, nitrogen oxides, sulphur dioxides, and chlorides which gives the impact to the human health as well [37]. Particulate matter formation also contributed to the human health endpoint category which derives greatly from emissions of sulphur dioxide resulting mainly from production and manufacturing of aluminum products [38]. Though, the endpoint effects on the assets were mostly contributed by fossil exhaustion fundamentally derived from utilization of coal, petroleum gas, and oil to a great extent utilized in the creation of aluminum. The fossil fuel and metal exhaustion mirrors ecological worries regarding asset utilization, which thusly energizes the execution of reuse. By reusing aluminum, decreases in material and vitality utilization are foreseen in contrast with essential creation [35].

On the other hand, the CR has a larger impact of up to 66.04% for sum up of endpoints value compared to DR-HPF process. The CR melting technique includes the unit processes of decorating, shredding, re-melting, and secondary ingot casting [38]. The CR process is an energy intensive method, and the total energy efficiency is insignificant [35,39]. In addition, matters of concern regarding CR include the lasting material failures amid re-liquefying as a result of oxidation. This viewpoint is especially applicable for chips or light-measure scraps which, having a high surface zone to-volume proportion, will in general buoy on the outside of the liquefaction, causing a noteworthy oxidation that can be as high as 15–20% [40]. Castro et al. [41], Amini et al. [42], and Chiba et al. [43] featured three sorts of losses amid metal reusing: material, quality, and weakening misfortunes. Material failures incorporate physical failures amid scrap readiness procedures and dissolving misfortunes, for example, oxidation failures just as build ups and slag squander that are landfilled. Quality failures happen when the quality (which means structure) of the created auxiliary metal does not coordinate with the info material. Weakening failures happen when high immaculateness metal is required to bring down the contaminant (residual) fixation to as far as possible for the target alloy.

Furthermore, the current practices of CR aluminum in most industries are using a melting technique to produce a secondary ingot by controlling the composition of alloy to match the standardized grades. Consequently, metal losses need to be balanced with primary aluminum. Unfortunately, essential aluminum creation is a standout amongst the most energy consuming materials, requiring by and large 66MJ of energy per kg in 2012, of which 80% is power in the Hall-Héroult process [44]. The greenhouse gas (GHG) outflows related with the 2007 worldwide aluminum cycle represent 0.45 Gigatons CO_2_ eq., or around 1% of the worldwide GHG emissions [39]. Smelting and other essential generation-related procedures (mining, refining, and creation of anodes for smelting) together are in charge of over 90% of the all-out emanations. The energy devoured in any phase in the existence cycle of the creation chain significantly affects the related stock of discharges and ecological effects on account of expansive contrasts in power age. The most imperative discharge source is circuitous emanations (65% of the aggregate), predominately happening from power creation, trailed by procedure outflows (18%) and petroleum products (17%) [44]. Since the removal of alloying element during re-melting is very difficult or problematic for most elements, DR approach by utilizing hot press forging of aluminum chips can provide significant environmental benefits, mainly by avoiding metal losses during re-melting. By avoiding remelting, significant amounts of both energy and material can be saved and, in consequence, the overall environmental impact due to aluminium usage can be significantly reduced.

### 3.3. Global Warming Potential (GWP)

Climate change is the category which handles all substances that contribute to the changing of the global climate. The idea of carbon impression is generally direct, as it represents all greenhouse gas (GHG) discharges over an item’s life cycle. Most methodologies depend on life cycle evaluation (LCA) as an all-encompassing ecological bookkeeping strategy for estimating and contrasting the natural results of an item or an administration for an incredible duration cycle. Global warming potential (GWP) is the quantification of the warming effect a substance given in carbon dioxide, CO_2_-equivalents. When doing the impact assessment, the result for the climate change category is given in GWP which unit is kg of CO_2_-equivalents. In spite of the fact that carbon dioxide, to a great extent discharged from the ignition of non-renewable energy sources, is the most noteworthy GHG outflow in terms of both mass and impact, we perceive that the carbon impression by and by assesses the commitments of a container of GHG gases that add to an unnatural weather change. Table 14 and Table 15 demonstrate the outcomes for related gasses to the aluminum creation process that is being collected into a solitary unit as far as their effect as carbon dioxide counterparts from the [1] for DR-HPF process and CR of aluminum respectively. Five related gasses are carbon dioxide (CO_2_), methane (CH_4_), sulphur dioxide (SO_2_), nitrous oxide (N_2_O), and perflurocarbon (PFC-14). CO_2_ gasses were mostly released in both routes whereas, N_2_O and PFC-14 are not applicable from this process.

The global warming potential (GWP) of a DR using hot press forging routes gives a significant environmental impact where it is reduced up to 69.2% as compared to the conventional (CR) using melting approach. By looking at the outcomes, it is conceivable to see that the DR utilizing hot press manufacturing system prompts generous CO_2_ outflows decrease. This is because of two-fold preferred standpoint that the strong state reuse system offers in diminished procedure energy/emanations and generous material failure decreases (with the strong state reuse showing changeless material failures). In fact, the outcomes expect equivalent mechanical properties as talked about in LCIA for the items obtained by the DR-HPF courses and their individual reference as-recieved items. Upgraded mechanical properties can prompt further effect decreases. The natural effect of the hot press producing process is exceedingly controlled by electrical energy utilization per unit of mass. To decrease this value, procedures could be the generation of substantial or various little items, shorter sintering cycles, and improved energy productivity of the procedure itself. Material losses that have been avoided and successfully decreasing energy utilization, as discussed in LCIA, are the real components clarifying the critical effect decreases acquired. This is a legitimate for scrap types with a high surface-to-volume proportion because of the critically maintained strategic distance from material losses brought about by substantial oxidation in the CR course. Duflou et al. [23] agreed with these results and stated that the major maintained a strategic distance from effect results is from the end of material failures in the strong state reuse courses. The affectability investigation in the examination plainly represents that the advantages of strong state reuse are most blunt for light check generation scrap bringing about high material failures amid re-liquefication. This paper demonstrates the significant environmental benefits that are possible by utilizing imaginative reuse systems rather than the re-softening methodology. Although it is still primarily established via execution in investigational settings, the possibility for substantial environmental impact reductions obtainable by DR approaches for scrap production can be evidently established. This analysis vindicated the significance of the hot press forging process in bringing environmental benefits as compared to CR.

In this paper, the environmental benefits of a DR AA6061 using hot press forging process and CR incorporating melting/casting were quantified and compared the for both recycling routes. CR presents a larger impact of up to 66.04% for all endpoints compared to the DR-HPF process. Meanwhile, six endpoints for every impact category show that the most substantial impact values for both routes are climate change (human health) and fossil depletion, followed by particulate matter, change of climate (ecosystem), human toxicity, and metal depletion. The global warming potential of a DR-HPF routes can offer significant environmental impact where it is reduced up to 69.2% by exhibited 34.97 kgCO_2_-eq/kg as contrasted to the traditional (melting) routes which are 113.53 kgCO_2_-eq/kg. This analysis vindicated the hot press forging procedure given the noteworthy ecological advantages in contrast to the CR.

## 4. Conclusions and Outlook

This paper depicts the outcomes of the study on the influences of operating temperature, holding time with constant force on the UTS, ETF, and GWP of recycling AA6061 chips by utilizing hot press forging process. The result per ANOVA analysis shows that the operating temperature is the most substantial aspect affecting the response variables investigated. This developed RSM model can now be used for analysis and predict the UTS, elongation, and hardness for recycling aluminum chip by the hot press forging process. Response surface optimization shows that the optimal combination of hot press forging parameters is 530 °C for 82.19 min for operating temperature and holding time respectively. In conjunction to this, the environmental benefits of a DR-HPF (530 °C and 82.19 min optimal suggested from RSM analysis) and CR (melting) were quantified and compared for both recycling routes. CR gives larger impact up to 66.04% for all endpoints compared to DR-HPF process. Meanwhile, six endpoints per impact category give the most significant impact values for both routes—these are climate change (human health) and fossil depletion; followed by particulate matter, climate change (ecosystem), human toxicity, as well as metal depletion. The global warming potential of a DR-HPF route offers significant environmental impact where it is reduced up to 69.2% by exhibited 34.97 kgCO_2_-eq/kg as compared to the conventional (melting) routes is 113.53 kgCO_2_-eq/kg. Despite the fact that it is uncertain for the most part based on perceptions in trial settings, the potential for huge ecological effect decreases offered by the DR strategies for generation scrap can be unmistakably illustrated. This examination underlines that the hot press manufacturing process clearly presents ecological advantages when contrasted with CR. Improved energy effectiveness is at present the focal point of progressing research which permits further effect decreases. This paper offers points of view for mechanical advancement of strong state reuse forms as environmentally-friendly alternatives to present liquefication-based practices. The environmental impact of hot press forging is highly determined by the electrical energy consumption per unit mass. The recommendation for further study is to reduce the value of energy consumption by pursuing strategies to produce large scale or multiple small products for improved energy efficiency of the process itself.

## Figures and Tables

**Table 1:** Experimental design parameters and levels.

Factor	Parameter	Notation	Unit	Levels		
				(−1)	(0)	(+1)
A	Operating temperature	T_s_	°C	430	480	530
B	Holding time	t_s_	minutes	60	90	120

**Table 2:** Main inventories data and sources for each process and materials.

Process	Details	Source
Chemical degreasing/drying	Datasets incorporate the energy utilization just as working materials expected to work degreasing baths.	Ecoinvent database v3. 1 2014
Hot press forging	Heating up to 430, 480, 530 °C Holding at 430, 480, 530 °C, 350 kN for 60, 90, 120 min Standby mode by hold at 350 kN	Measured energy consumption per unit mass.

**Table 3:** Hot press forging settings and measurement.

Process	Setting
Furnace	240 volts
Forging	415 volts
Heating	5.337 kWh
Hot Press Forging Process	6.683 kWh
Standby Mode	0.592 kWh

**Table 4:** Main life cycle inventory data and sources for each process.

Route	Process	Details	Source
Direct recycling (hot press forging)	Chemical degreasing/drying	Datasets incorporate the energy utilization just as working materials expected to work degreasing baths.	Ecoinvent database v3.1 2014
	Hot press forging	Heating up to 530 °C Holding at 530 °C, 35 kN for 82.19 min Standby mode by hold at 35 kN force	Measured energy consumption per unit mass.
Conventional recycling (melting)	Secondary Al production from new scrap	Melting, dross recycling and casting are incorporated. Mg loss as alloying element is also included.	Ecoinvent database v3.1 2014
	Primary AA6061	The alloying elements’ concentration midpoint for alloy limit of tolerance.	Ecoinvent database v3.1 2014
	Alloying elements (Mn, Mg, Si, Cu)	Extraction of resource up to the factory gate consisting of infrastructure.	Ecoinvent database v3.1 2014

**Table 5:** Design of experimental matrix in hot press forging process.

Std. Run	Factors (Coded)		Factors (Actual)	
	*C*_1_	*C*_2_	*A*—Temperature (°C)	*B*—Holding Time (minutes)
1	−1	−1	430	60
2	+1	−1	530	60
3	−1	+1	430	120
4	+1	+1	530	120
5	−1	0	430	90
6	+1	0	530	90
7	0	−1	480	60
8	0	+1	480	120
9	0	0	480	90
10	0	0	480	90
11	0	0	480	90

**Table 6:** Experimental results on UTS, ETF, and GWP.

Std.Run	Factor 1 A: Operating Temperature, T_s_ (°C)	Factor 2 B: Holding Time, t_s_ (minute)	Ultimate Tensile Strength, UTS (MPa)	Elongation to Failure, ETF (%)	Global Warming Potential, GWP (kg CO_2_-eq/kg)
1	480.00	120.00	90.42	3.23	36.12
2	530.00	90.00	240.27	9.5	36.86
3	430.00	90.00	16.91	1.04	25.38
4	430.00	120.00	28.44	2.03	30.23
5	530.00	120.00	266.78	16.13	42.19
6	430.00	60.00	14.97	0.09	19.8
7	480.00	90.00	72.84	2.27	30.65
8	480.00	60.00	48.11	0.13	25.49
9	530.00	60.00	230.55	6.86	31.28
10	480.00	90.00	79.07	1.06	30.54
11	480.00	90.00	72.72	1.95	30.01

**Table 7:** ANOVA table for ultimate tensile strength, UTS.

Source	Sum of Squares	df	Mean Square	*f*-Value	Prob > *f*	
Model	87,407.86	5	17,481.57	172.65	<0.0001	significant
A-Operating Temperature	76,451.37	1	76,451.37	755.05	<0.0001	
B-Holding Time	2098.51	1	2098.51	20.73	0.0061	
AB	129.5	1	129.5	1.28	0.3094	
A^2^	7613.84	1	7613.84	75.2	0.0003	
B^2^	79.36	1	79.36	0.78	0.4165	
Residual	506.26	5	101.25			
Lack of Fit	479.88	3	159.96	12.13	0.0771	not significant
Pure Error	26.38	2	13.19			
Cor Total	87,914.13	10				
Std. Dev.	10.06		R^2^	0.9942		
Mean	107.39		Adj. R^2^	0.9885		
C.V. %	9.37		Pred. R^2^	0.9417		
PRESS	5127.82		Adeq. Precision	35.411		

**Table 8:** ANOVA table for ultimate tensile strength, UTS (reduced).

Source	Sum of Squares	df	Mean Square	*f*-Value	Prob > *f*	
Model	87,198.99	3	29,066.33	284.51	<0.0001	significant
A-Operating Temperature	76,451.37	1	76,451.37	748.34	<0.0001	
B-Holding Time	2098.51	1	2098.51	20.54	0.0027	
A^2^	8649.11	1	8649.11	84.66	<0.0001	
Residual	715.13	7	102.16			
Lack of Fit	688.75	5	137.75	10.44	0.0897	not significant
Pure Error	26.38	2	13.19			
Cor Total	87,914.13	10				
Std. Dev.	10.11		R^2^	0.9919		
Mean	107.39		Adj. R^2^	0.9884		
C.V. %	9.41		Pred. R^2^	0.9749		
PRESS	2203.15		Adeq. Precision	43.177		

**Table 9:** ANOVA table for elongation to failure, ETF.

Source	Sum of Squares	df	Mean Square	*f*-Value	Prob > *f*	
Model	239.9732	5	47.99465	48.72529	0.0003	significant
A-Operating Temperature	143.3748	1	143.3748	145.5575	<0.0001	
B-Holding Time	34.12935	1	34.12935	34.64892	0.0020	
AB	13.43223	1	13.43223	13.63671	0.0141	
A^2^	42.21985	1	42.21985	42.86259	0.0012	
B^2^	0.614148	1	0.614148	0.623497	0.4655	
Residual	4.925024	5	0.985005			
Lack of Fit	4.138824	3	1.379608	3.50956	0.2296	not significant
Pure Error	0.7862	2	0.3931			
Cor Total	244.8983	10				
Std. Dev.	0.99		R^2^	0.9799		
Mean	4.03		Adj. R^2^	0.9598		
C.V. %	24.65		Pred. R^2^	0.8267		
PRESS	42.45		Adeq. Precision	21.246		

**Table 10:** ANOVA table for elongation to failure, ETF (reduced).

Source	Sum of Squares	df	Mean Square	*f*-Value	Prob > *f*	
Model	225.9269	3	75.30895	27.78724	0.0003	significant
A-Operating Temperature	143.3748	1	143.3748	52.90194	0.0002	
B-Holding Time	34.12935	1	34.12935	12.59293	0.0094	
A^2^	48.42269	1	48.42269	17.86684	0.0039	
Residual	18.9714	7	2.7102			
Lack of Fit	18.1852	5	3.637039	9.252199	0.1004	not significant
Pure Error	0.7862	2	0.3931			
Cor Total	244.8983	10				
Std. Dev.	1.65		R^2^	0.9225		
Mean	4.03		Adj. R^2^	0.8893		
C.V. %	40.89		Pred. R^2^	0.7192		
PRESS	68.76		Adeq. Precision	14.653		

**Table 11:** ANOVA table for global warming potential, GWP.

Source	Sum of Squares	Degree of Freedom	Mean Square	*f*-Value	Prob > *f*	
Model	373.5812	2	186.7906	1474.855	<0.0001	significant
A-Operating Temperature	203.2344	1	203.2344	1604.691	<0.0001	
B-Holding Time	170.3468	1	170.3468	1345.018	<0.0001	
Residual	1.013202	8	0.12665			
Lack of Fit	0.779002	6	0.129834	1.108741	0.5455	not significant
Pure Error	0.2342	2	0.1171			
Cor Total	374.5944	10				
						
Standard Deviation	0.36		R^2^	0.9973		
Mean	30.78		Adjusted R^2^	0.9966		
Coefficient of variation %	1.16		Predicted R^2^	0.9958		
Predicted residual error of sum of square	1.56		Adequate Precision	119.970		

**Table 12:** Suggested solution for optimum responses.

Solutions	Operating Temperature	Holding Time	UTS	ETF	GWP	Desirability	
1	530.00	82.19	241	10.2093	35.2106	0.642	Selected
2	529.53	85.59	241	10.3546	35.7597	0.630	
3	529.98	94.16	248.376	11.1556	37.3333	0.511	

**Table 13:** Confirmation test and comparison with predicted value.

Output	Details	Confirmation Test	
		1	2
	Operating Temperature, T_s_ (°C)	530	530
	Holding Time, t_s_ (minutes)	82.19	82.19
Ultimate Tensile Strength, UTS (MPa)	Experimental	246.50	252.56
	Prediction	241.00	241.00
	Error (%)	2.28	4.81
Elongation to Failure, ETF (%)	Experimental	11.54	12.25
	Prediction	10.21	10.21
	Error (%)	13.02	19.98
Global Warming Potential, GWP (kg CO_2_-eq/kg	Experimental	34.97	36.21
	Prediction	35.21	35.21
	Error (%)	0.69	2.84

**Table 14:** Results for related gasses being aggregated into a single unit in terms of their impact as carbon dioxide equivalents for DR-HPF process.

No.	Element	Designation	LCA Value		GWP Coef.		Total GWP
1	Carbon dioxide	CO_2_	33.42566	X	1	=	33.43
2	Methane	CH_4_	0.06865	X	21	=	1.44
3	Nitrous oxide	N_2_O	n/a	X	310	=	0.00
4	Sulphur dioxide	SO_2_	0.0000145	X	7400	=	0.11
5	PFC-14	CF_4_	n/a	X	6500	=	0.00
*Total GWP* (kg CO_2_-eq/kg)							*34.97*

**Table 15:** Results for related gasses being aggregated into a single unit in terms of their impact as carbon dioxide equivalents for CR process.

No.	Element	Designation	LCA Value		GWP Coef.		Total GWP
1	Carbon dioxide	CO_2_	106.6232	X	1	=	106.62
2	Methane	CH_4_	0.3222	X	21	=	6.77
3	Nitrous oxide	N_2_O	n/a	X	310	=	0.00
4	Sulphur dioxide	SO_2_	0.0000192	X	7400	=	0.14
5	PFC-14	CF_4_	n/a	X	6500	=	0.00
*Total GWP* (kg CO_2_-eq/kg)							*113.53*

## Nomenclature

A	first factor or input variable investigated–Operating temperature (ºC)
Adeq. precision	adequate precision
Adj. R2	adjusted R2
B	second factor or input variable investigate–Holding time (minutes)
C.V. %	coefficient of variation
Cor. total	totals of all information corrected for the mean
CR	conventional recycling
d.f.	degrees of freedom
DR	direct recycling
DR-HPF	direct recycling hot press forging
ECAP	equal channel angular pressing
ETF	elongation to failure (%)
GHG	greenhouse gasses
GWP	global warming potential (kg CO_2_-eq/kg)
LCA	life cycle assessment
OFAT	one factor at a time
Pred. R2	predicted R2
PRESS	predicted residual error sum of squares
Prob. > F	proportion of time or probability you would expect to get the stated F value
R2	coefficient of determination
SPS	spark plasma sintering
Std. Dev.	square root of the residual mean square
UTS	ultimate tensile strength (MPa)
